# Flux Growth and Superconducting Properties of (Ce,Pr)OBiS_2_ Single Crystals

**DOI:** 10.3389/fchem.2020.00044

**Published:** 2020-02-04

**Authors:** Masanori Nagao, Akira Miura, Daisuke Urushihara, Yuki Maruyama, Yosuke Goto, Yoshikazu Mizuguchi, Chikako Moriyoshi, Yoshihiro Kuroiwa, Yongming Wang, Satoshi Watauchi, Toru Asaka, Yoshihiko Takano, Kiyoharu Tadanaga, Isao Tanaka

**Affiliations:** ^1^Center for Crystal Science and Technology, University of Yamanashi, Kofu, Japan; ^2^Faculty of Engineering, Hokkaido University, Sapporo, Japan; ^3^Division of Advanced Ceramics, Nagoya Institute of Technology, Nagoya, Japan; ^4^Department of Physics, Tokyo Metropolitan University, Hachioji, Japan; ^5^Department of Physical Science, Hiroshima University, Hiroshima, Japan; ^6^Creative Research Institution, Hokkaido University, Sapporo, Japan; ^7^MANA, National Institute for Materials Science, Tsukuba, Japan

**Keywords:** BiS_2_ superconductor, flux growth, TEM, single crystals, XAFS

## Abstract

Ce_1−*x*_Pr_*x*_OBiS_2_ (0. 1 ≤ *x* ≤ 0.9) single crystals were grown using a CsCl flux method. Their structural and physical properties were examined by X-ray diffraction, X-ray absorption, transmission electron microscopy, and electrical resistivity. All of the Ce_1−*x*_Pr_*x*_OBiS_2_ single crystals with 0.1 ≤ *x* ≤ 0.9 exhibited tetragonal phase. With increasing Pr content, the *a*-axis and *c*-axis lattice parameters decreased and increased, respectively. Transmission electron microscope analysis of Ce_0.1_Pr_0.9_OBiS_2_ (*x* = 0.9) single crystal showed no stacking faults. Atomic-resolution energy dispersive X-ray spectrometry mapping revealed that Bi, Ce/Pr, O, and S occupied different crystallographic sites, while Ce and Pr randomly occupied the same sites. X-ray absorption spectra showed that an increase of the Pr ratio increased the ratio of Ce^4+^/Ce^3+^. All of the Ce_1−*x*_Pr_*x*_OBiS_2_ crystals showed superconducting transition, with a maximum transition temperature of ~4 K at *x* = 0.9.

## Introduction

Flux synthesis has been utilized widely for the synthesis of solid-state materials (Oishi et al., 2004; Akira et al., 2019), especially for discovering compounds with complex structures (DiSalvo and Clarke, 1996; Bugaris and zur Loye, 2012) Nonetheless, an interesting feature of synthesis using a flux, in addition to structural determination, is to explore various properties (Yamane et al., 1997; Mizuno et al., 2014; Miura et al., 2016; Chiang et al., 2018). Superconducting materials also can be synthesized by the flux method, and the properties of the synthesized products can be controlled by the synthesis condition (Nagao, 2017). For example, we very recently found that Sm(O,F)BiS_2_ single crystals synthesized by KCl-KI exhibited superconductivity (Kinami et al., 2019), in contrast to the non-superconducting Sm(O,F)BiS_2_ powder and crystals synthesized using CsCl-KCl flux reported previously (Thakur et al., 2015). Thus, flux synthesis under different experimental conditions could be a promising approach for discovering new superconducting materials.

BiS_2_-based layered compounds, composed of alternate BiS_2_ and RO layers (R: rare earth elements), have been studied widely as a new family of superconducting and thermoelectric materials (Mizuguchi, 2019). Superconductivity can be induced by carrier doping (Mizuguchi et al., 2012) and in-plane chemical pressure (Mizuguchi et al., 2015). Carrier doping can be achieved by doping of F^−^ into O^2−^ sites or by valence fluctuation of rare earths, such as Ce^3+^ and Ce^4+^ (Nagao et al., 2016; Tanaka et al., 2017; Miura et al., 2018; Hanada et al., 2019). The induction of carrier by Ce valence fluctuation can be formulated as below;

$$\begin{array}{l} {\text{Ce}}^{3+}={\text{Ce}}^{4+}+\;{\text{e}}^{-} \end{array}$$

To present, various ROBiS_2_ and R(O,F)BiS_2_ compounds have been synthesized using flux methods (Nagao et al., 2013, 2014a, 2015, 2016, 2019; Miura et al., 2014, 2015, 2018; Sagayama et al., 2015; Tanaka et al., 2017; Hanada et al., 2019). Recently, we reported the synthesis and superconducting properties of (Ce,Pr)OBiS_2_. (Ce,Pr)OBiS_2_ powders showed a maximum superconducting temperature of ~2.4 K, when the ratio of Ce/Pr was the same, but these were a mixture of tetragonal and monoclinic phases (Miura et al., 2018). The monoclinic phase, slightly distorted from the tetragonal phases, affects the dimensionality of the conduction path of the BiS_2_ layer significantly. The monoclinic distortion from the tetragonal cell, a/b ratio, is only ~0.001, which can be detected only by a highly monochromatic synchrotron X-ray. To clarify the origin of the superconductive phase, we conducted flux growth of single crystals and found that the tetragonal phase of Ce_0.5_Pr_0.5_OBiS_2_ is superconducting. However, the investigation of single crystals in (Ce,Pr)OBiS_2_ is limited with Ce_0.5_Pr_0.5_OBiS_2_. The flux growth of (Ce,Pr)OBiS_2_ single crystals with various Ce/Pr ratios would provide an opportunity for discovering new superconductors.

In this study, we successfully grew Ce_1−*x*_Pr_*x*_OBiS_2_ superconducting single crystals with different Ce/Pr ratios (x = 0.1, 0.3, 0.5, 0.7, 0.9) using a CsCl flux. The crystals with high Pr contents showed superconducting properties with a maximum transition temperature of ~4 K, which could not be observed in powder samples synthesized without using the flux (Miura et al., 2018). The single crystals of Ce_1−*x*_Pr_*x*_OBiS_2_ were characterized by X-ray diffraction and also X-ray absorption fine spectroscopy for the Ce and Pr valence and superconducting properties. Transmission electron microscope analysis revealed direct evidence for mixed Ce/Pr sites and other elements with specific crystallographic sites.

## Experimental

Single crystals of Ce_1−*x*_Pr_*x*_OBiS_2_ (0.1 ≤ *x* ≤ 0.9) were grown using CsCl flux (Miura et al., 2018). The raw materials of Ce_2_S_3_ (99.9%: Mitsuwa Chemicals), Pr_2_S_3_ (99.9%: Mitsuwa Chemicals), Bi_2_O_3_ (99.9%: Kojyundo Chemical Lab.), Bi_2_S_3_ (99.9%: Mitsuwa Chemicals) were weighed to have a nominal composition of Ce_1−*x*_Pr_*x*_OBiS_2_ (0.1 ≤ *x* ≤ 0.9). The mixture of the raw materials (0.8 g) and CsCl flux (5.0 g) were ground by using a mortar, and then sealed into an evacuated quartz tube. The quartz tube was heated at 950°C for 10 h, followed by cooling to 650°C at a rate of 1°C/h, then the sample was cooled down to room temperature in the furnace. The heated quartz tube was opened in air, and the obtained materials were washed and filtered by distilled water for removing the CsCl flux.

The compositional ratio of the single crystals was evaluated by energy dispersive X-ray spectrometry (EDS) (Bruker, Quantax 70) associated with the observation of the microstructure by using scanning electron microscope (SEM) (Hitachi High-Technologies, TM3030). The obtained compositional values were normalized using S = 2.00, with Ce, Pr, and Bi measured to a precision of two decimal places. After that, the Pr composition is normalized by the total Pr and Ce content. Identification and orientation of the grown crystals were performed by X-ray diffraction (XRD) using Rigaku MultiFlex with CuKα radiation. Crystal system and lattice parameters of (Ce,Pr)OBiS_2_ single crystals were evaluated from synchrotron powder X-ray diffraction measurements using crushed single crystals powder. Synchrotron powder X-ray diffraction measurements were performed at 150 K in SPring-8 using the BL02B2 beamline with the approval of 2018A0074. Local structure and elemental distribution of Ce_0.1_Pr_0.9_OBiS_2_ (*x* = 0.9) single crystal along the *c*-axis were observed by scanning transmission electron microscope with energy dispersive X-ray spectrometry (STEM-EDS) (JEOL JEM-ARM200F).

The valence state of the Cerium and Praseodymium component in the obtained single crystals was estimated by X-ray absorption fine spectroscopy (XAFS) analysis of Ce-*L*_3_ and Pr-*L*_3_ edges using an Aichi XAS beamline with a synchrotron X-ray radiation (BL11S2: Experimental No.201801025). For XAFS sample, the obtained single crystals were grinded and mixed with boron nitride (BN) powder, pressed into a pellet of 4 mm diameter.

The Resistivity-temperature (ρ-*T*) characteristics of the obtained single crystals were measured by the standard four-probe method with a constant current density (*J*) mode using physical property measurement system (Quantum Design; PPMS DynaCool). The electrical terminals were fabricated by silver paste. And, ρ–*T* characteristics in the temperature range of 0.25–15 K were measured with an adiabatic demagnetization refrigerator (ADR) option for PPMS. The magnetic field applied for operating the ADR was 3 T at 1.9 K; subsequently, it was removed. Consequently, the temperature of sample decreased to ~0.25 K. The measurement of ρ–*T* characteristics was started at the lowest temperature (~0.25 K), which was spontaneously increased to ~15 K. The superconducting transition temperature (*T*_c_) was estimated from the ρ–*T* characteristics. The transition temperature corresponding to the onset of superconductivity ($T_{\text{c}}^{\text{onset}}$) is defined as the temperature at which deviation from linear behavior is observed in the normal conducting state in the ρ–*T* characteristics. The zero resistivity ($T_{\text{c}}^{\text{zero}}$) is determined as the temperature at which resistivity is below 10 μΩ cm. The ρ–*T* characteristics of Ce_0.1_Pr_0.9_OBiS_2_ (*x* = 0.9) single crystal under a magnetic field (*H*) parallel to the *c*-plane with range of 0.1–9.0 T and the *c*-axis with range of 0.1–0.3 T were measured in the temperature range of 2.0–10.0 K. We measured the angular (θ) dependence of resistivity (ρ) in the flux liquid state under various magnetic fields (*H*) and calculated superconducting anisotropy (γ_s_) using the effective mass model (Blatter et al., 1992; Iwasaki et al., 1995).

## Results and Discussion

**Figure 1:**
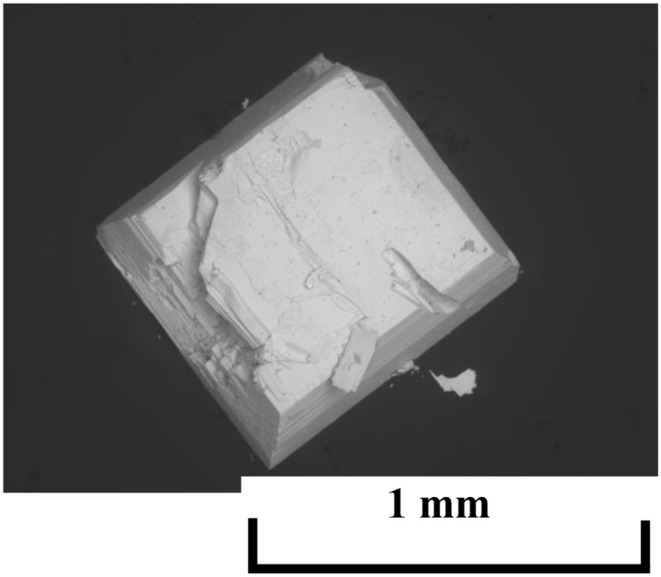
Typical SEM image of (Ce,Pr)OBiS_2_ single crystal.

Figure 1 shows a typical SEM image of a (Ce,Pr)OBiS_2_ single crystal. The single crystals obtained had a plate-like shape, size of around 1.0 mm, and thickness of 30–50 μm. Qualitative analysis by EDS showed that the estimated atomic ratios of Bi and S elements in the single crystals were almost stoichiometric, with Bi:S = 1.01 ± 0.06. On the other hand, Cs and Cl from the flux were not detected in the single crystals with a minimum sensitivity limit of ~1 wt%. Analytical Pr contents of the single crystals obtained with various Ce/Pr ratios (*x*) are shown as Table 1. The analyzed values were almost in the same ratio as the nominal compositions.

**Table 1:** Nominal and analytical Pr ratios.

**Pr contents**	**Nominal (*x*)**	**0.1**	**0.3**	**0.5**	**0.7**	**0.9**
	Analytical (Ce + Pr = 1.00)	0.091	0.32	0.48	0.71	0.90

*The analytical Pr ratio were calculated from the total of Ce and Pr*.

**Figure 2:**
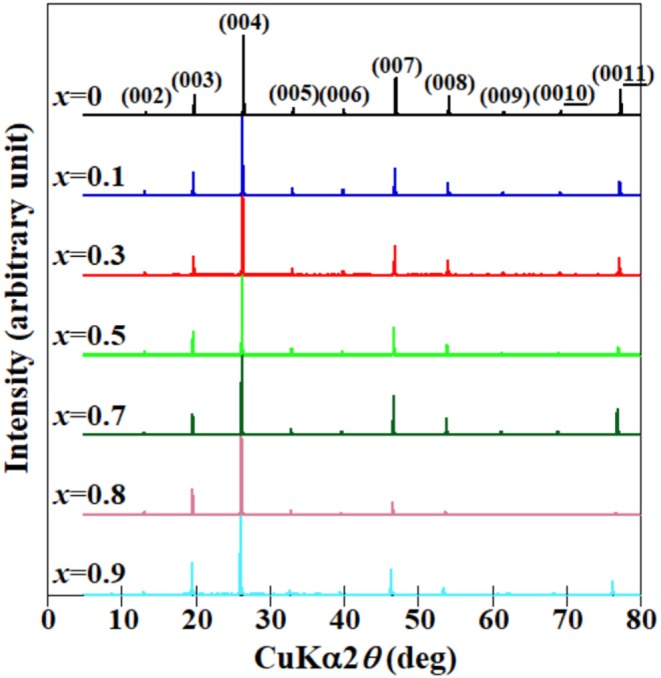
XRD patterns of well-developed plane of (Ce,Pr)OBiS_2_ with 0.1 ≤ *x* ≤ 0.9 and CeOBiS_2_ (*x* = 0) (Nagao et al., 2016) single crystals.

**Figure 3:**
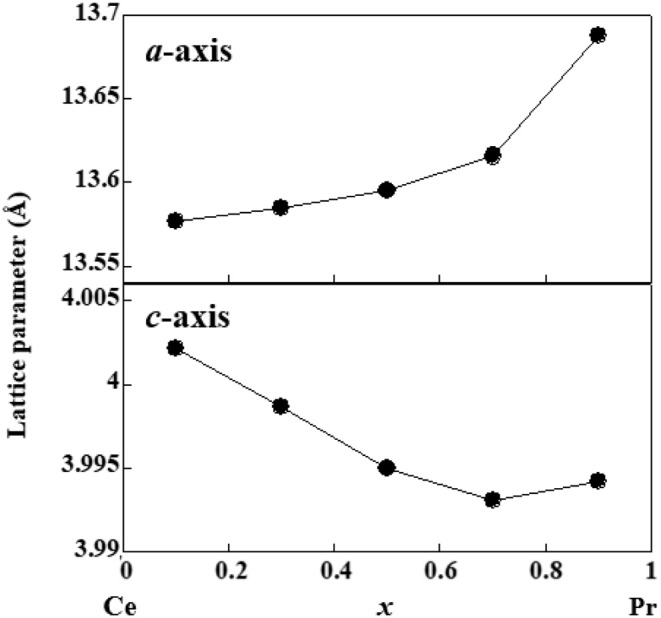
Lattice parameters of *a* and *c*-axes of (Ce,Pr)OBiS_2_ single crystals with 0.1 ≤ *x* ≤ 0.9 at 150 K.

Figure 2 shows the XRD patterns of a well-developed plane in the obtained Ce_1−*x*_Pr_*x*_OBiS_2_ (0.1 ≤ *x* ≤ 0.9) single crystals. The appearance of only 00*l* diffraction peaks indicates that the *c*-plane is well-developed. This is a typical feature of BiS_2_-based single crystals (Nagao et al., 2013, 2014a, 2015, 2016; Miura et al., 2014, 2015, 2018; Sagayama et al., 2015; Hanada et al., 2019). Synchrotron powder X-ray diffraction measurements of the crushed single crystals at 150 K exhibited no splitting of 110 diffraction peaks, suggesting tetragonal phases. The lattice parameters are shown in Figure 3. The *a*-axis lattice parameters decreased with increased Pr contents (*x*), but increased slightly at *x* = 0.9. The lattice parameter of the *c*-axis increased with increased Pr contents (*x*).

**Figure 4:**
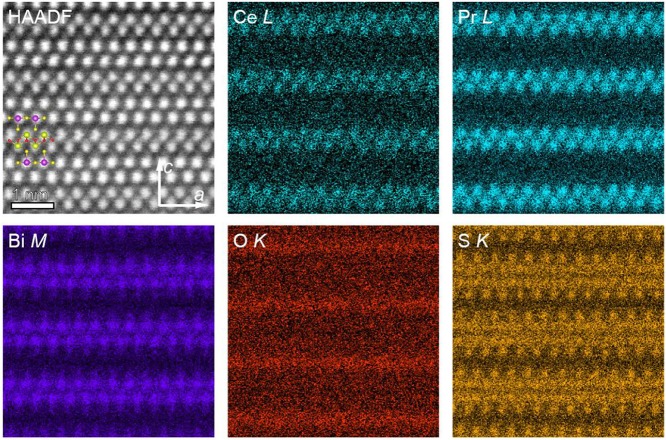
[010]-zone axis HAADF-STEM image and Ce, Pr, Bi, O, S elemental maps of (Ce,Pr)OBiS_2_ with *x* = 0.9 single crystal.

Direct observation of the stacking layers along the *c*-axis was performed by energy dispersive X-ray spectrometry mapping with a scanning transmission electron microscope (STEM-EDS). Figure 4 shows the high-angle annular-dark-field (HAADF) STEM image taken with the incident beam parallel to the [010] direction for (Ce,Pr)OBiS_2_ with *x* = 0.9 single crystal and also shows the corresponding elemental maps obtained using Ce-*L*, Pr-*L*, Bi-*M*, O-*K*, and S-*K* in the same area. No stacking faults were observed in the STEM image for a wide area. Atomic-resolution EDS mapping revealed that Bi, Ce/Pr, O, and S occupied different crystallographic sites, whereas Ce and Pr randomly occupied the same sites.

**Figure 5:**
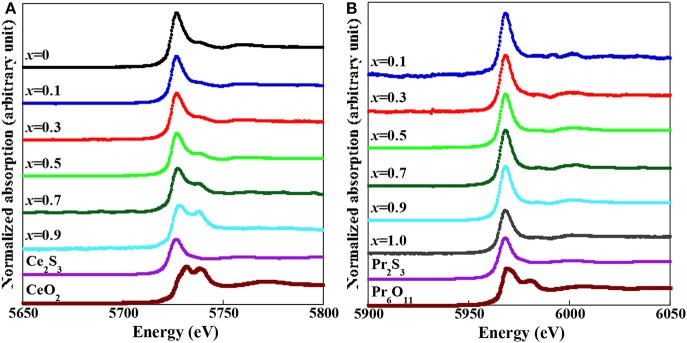
**(A)** Ce *L*_3_-edge, XAFS obtained at room temperature for (Ce,Pr)OBiS_2_ with 0.1 ≤ *x* ≤ 0.9 single crystals, CeOBiS_2_ (*x* = 0) single crystals, Ce_2_S_3_, and CeO_2_. **(B)** Pr L_3_-edge, XAFS obtained at room temperature for (Ce,Pr)OBiS_2_ with 0.1 ≤ *x* ≤ 0.9 single crystals, PrOBiS_2_ (*x* = 1) (Nagao et al., 2019) single crystals, Pr_2_S_3_, and Pr_6_O_11_.

We evaluated the valence state of Ce and Pr in the obtained (Ce,Pr)OBiS_2_ single crystals by XAFS analysis. Figure 5 shows (a) Ce *L*_3_- and (b) Pr *L*_3_-edge absorption spectra of (Ce,Pr)OBiS_2_ single crystals with 0 ≤ *x* ≤ 1.0 obtained by XAFS analysis at room temperature. The Ce *L*_3_-edge of the (Ce,Pr)OBiS_2_ single crystals showed a peak at around 5,727 eV, assigned as Ce^3+^, with is consistent with the other XAFS result for the trivalent electronic configuration (Ce^3+^) (Yaroslavtsev et al., 2010). Peaks around 5,731 and 5,738 eV evolved with increase of Pr content (*x*), and these peaks were assigned tetravalent electronic configuration (Ce^4+^) (Yamazaki et al., 2000). Therefore, the ratio of Ce^4+^ [Ce^4+^/(Ce^3+^ + Ce^4+^)] increased with increase of Pr content (*x*), suggesting that the valence state of Ce fluctuated in the (Ce,Pr)OBiS_2_ single crystals. On the other hand, the Pr *L*_3_-edge of the (Ce,Pr)OBiS_2_ single crystals showed a peak at ~5,968 eV, which can be assigned as Pr^3+^. This is consistent with the other XAFS results for the trivalent electronic configuration (Pr^3+^) (Ku et al., 2002; Lin et al., 2003). The tetravalent electronic configuration (Pr^4+^) shows a signature peak at ~5,980 eV (Ku et al., 2002; Fujishiro et al., 2012), which was hardly observed in the (Ce,Pr)OBiS_2_ single crystals (Miura et al., 2018). Therefore, Pr valence in the (Ce,Pr)OBiS_2_ with 0.1 ≤ *x* ≤ 0.9 single crystals was only trivalent. The Ce valence in the (Ce,Pr)OBiS_2_ with 0.1 ≤ *x* ≤ 0.9 single crystals was a mixed state consisting of both trivalent (Ce^3+^) and tetravalent (Ce^4+^) valences.

**Figure 6:**
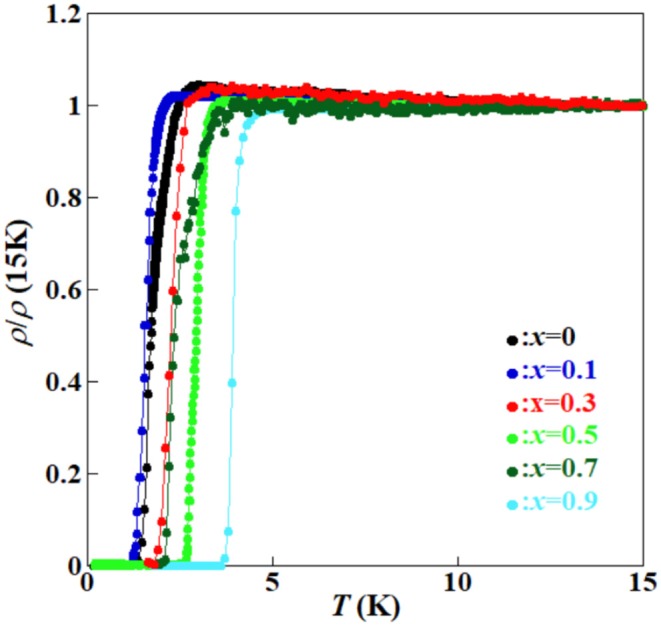
Temperature (*T*) dependence of resistivities normalized at 15 K [ρ/ρ(15 K)] for (Ce,Pr)OBiS_2_ with 0 ≤ *x* ≤ 0.9 single crystals in the temperature range of 0.25–15 K. The data of *x* = 0 was referred from the literature (Nagao et al., 2016).

**Figure 7:**
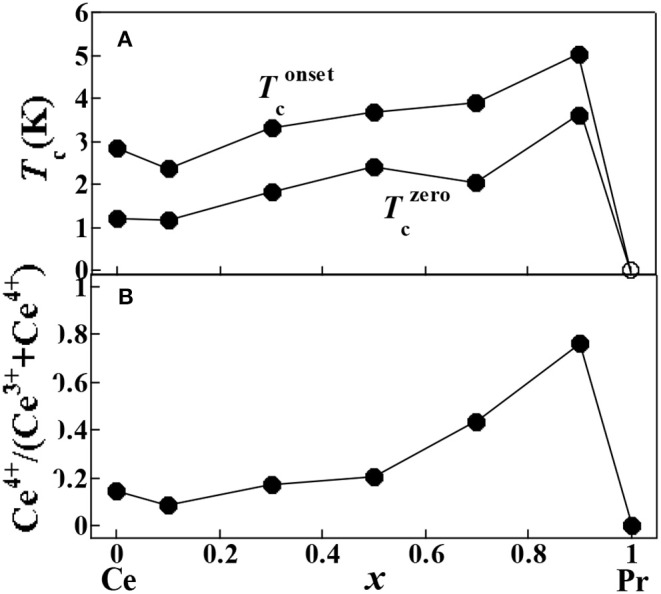
**(A)** The Pr contents (*x*) dependence of $T_{\text{c}}^{\text{onset}}$ and $T_{\text{c}}^{\text{zero}}$ for (Ce,Pr)OBiS_2_ with 0 ≤ *x* ≤ 0.9 and PrOBiS_2_ (*x* = 1) (Nagao et al., 2019) single crystals. **(B)** The Pr contents (*x*) dependence of Ce^4+^ ratio [Ce^4+^/(Ce^3+^ + Ce^4+^)] in (Ce,Pr)OBiS_2_ with 0 ≤ *x* ≤ 1.0 single crystals.

Figure 6 shows the temperature (*T*) dependence of resistivity normalized at 15 K [ρ/ρ(15 K)] for the (Ce,Pr)OBiS_2_ single crystals with 0 ≤ *x* ≤ 0.9 in the temperature range of 0.25–15 K. Above 5 K, decrease in temperature increases relative resistivity. The increases in relative resistivity are suppressed by higher Pr content. Figure 7A shows the Pr content (*x*) dependence of $T_{\text{c}}^{\text{onset}}$ and $T_{\text{c}}^{\text{zero}}$. $T_{\text{c}}^{\text{onset}}$, and $T_{\text{c}}^{\text{zero}}$. An increase in Pr content (*x*) increased the transition temperatures up to *x* = 0.9. In particular, (Ce,Pr)OBiS_2_ with *x* = 0.9 showed a highly superconducting transition temperature of ~4 K. The Pr content (*x*) dependence of the Ce^4+^ ratio [Ce^4+^/(Ce^3+^ + Ce^4+^)] in (Ce,Pr)OBiS_2_ single crystals with 0 ≤ *x* ≤ 1.0 showed similar tendency of the transition temperatures (Figure 7B).

**Figure 8:**
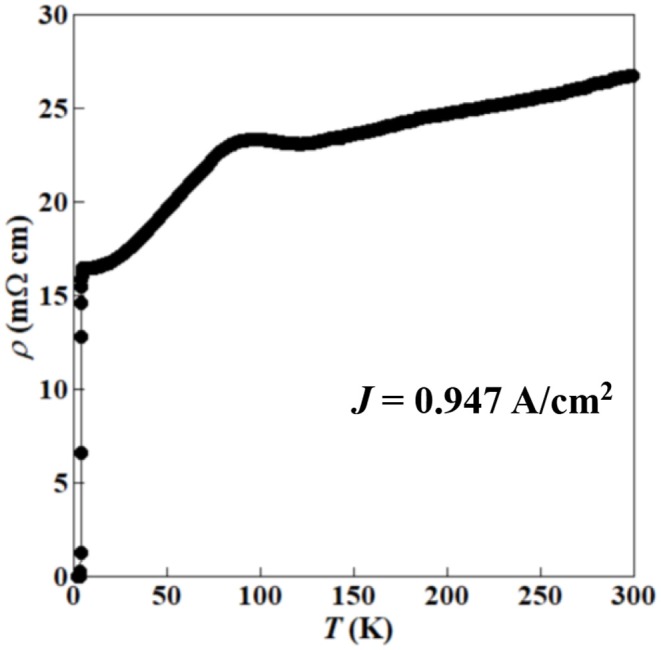
Resistivity–temperature (ρ-*T*) characteristics of (Ce,Pr)OBiS_2_ with *x* = 0.9 single crystal.

Figure 8 shows the ρ-*T* characteristics of (Ce,Pr)OBiS_2_ single crystal with *x* = 0.9. The resistivity at normal state shows almost metallic behavior although it shows an anomaly at 80–120 K. Similar anomalies of Nd(O,F)BiS_2_ and La(O,F)BiSe_2_ single crystal are reported in 60–140 K (Nagao et al., 2013) and 20–80 K (Nagao et al., 2014b), respectively. These anomalies are probably a common phenomenon of Bi*Ch*_2_-based (*Ch*:S,Se) superconductors although further investigation is required to reveal the origin of these anomalies.

**Figure 9:**
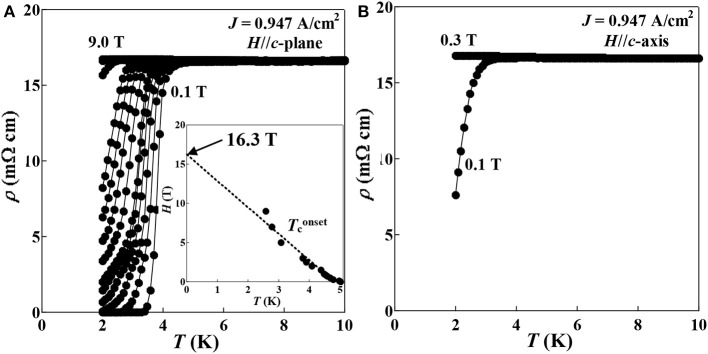
Temperature (*T*) dependence of resistivity (ρ) for (Ce,Pr)OBiS_2_ with *x* = 0.9 single crystal under the magnetic fields (*H*) parallel to the **(A)** *c*-plane (*H* = 0.1–9.0 T) and **(B)** *c*-axis (*H* = 0.1-0.3 T). The inset of **(A)** is the magnetic fields dependence of the $T_{\text{c}}^{\text{onset}}$ for (Ce,Pr)OBiS_2_ with *x* = 0.9 single crystal under the magnetic field (*H*) parallel to the *c*-plane (*H*//*c*-plane), and the lines are liner fits to the data.

Figure 9 shows the temperature dependence of resistivity for (Ce,Pr)OBiS_2_ single crystal with *x* = 0.9 below 10 K under the magnetic field (*H*) parallel to the *c*-plane (*H*//*c*-plane, *H* = 0.1–9.0 T) and to the *c*-axis (*H*//*c*-axis, *H* = 0.1–0.3 T). The superconducting transition was monotonically suppressed with increasing magnetic fields parallel to the *c*-plane. The magnetic fields dependence of the $T_{\text{c}}^{\text{onset}}$ under the magnetic field (*H*) parallel to the *c*-plane (*H*//*c*-plane) is plotted in the inset of Figure 9A. The linear extrapolation of $T_{\text{c}}^{\text{onset}}$ with *H*//*c*-plane approaches 16.3 T, which is estimated to be the maximum of upper critical field on the *c*-plane (*H*^//c−plane^c2). In a conventional (BCS-like) superconductor in the weak-coupling limit, the Pauli limit is calculated to be 9.22 T, derived from *H*_p_ = 1.84 *T*_c_, ($T_{\text{c}}^{\text{onset}}$ = 5.01 K) (Lu et al., 2013). Thus, the maximum of upper critical field in the *c*-plane (*H*^//c−plane^c2 = 16.3 T) is significantly higher than the Pauli limit (*H*_p_ = 9.22 T), indicating the possibility of an unconventional superconductor. In contrast to the superconductivity in the *c*-plane, its superconductivity in the applying magnetic fields perpendicular to the *c*-axis almost disappeared at 2 K with only 0.3 T (Figure 9B), suggesting its large superconducting anisotropy of (Ce,Pr)OBiS_2_ with *x* = 0.9. The upper critical field on the *c*-axis (*H*^//c−axis^c2) and the superconducting anisotropy (γ_s_) could not be estimated from $T_{\text{c}}^{\text{onset}}$, because the superconductivity was disappeared with low magnetic field (0.3 T).

**Figure 10:**
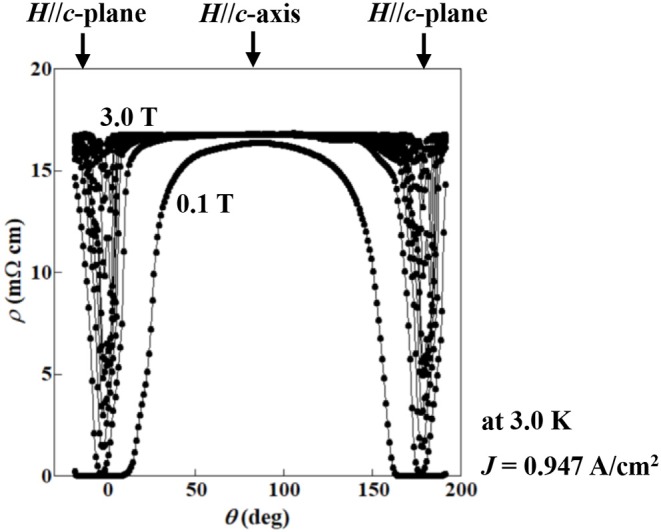
Angular (θ) dependence of resistivity (ρ) in flux liquid state at 3.0 K under various magnetic fields *H* = 0.1–3.0 T for (Ce,Pr)OBiS_2_ with *x* = 0.9 single crystal.

**Figure 11:**
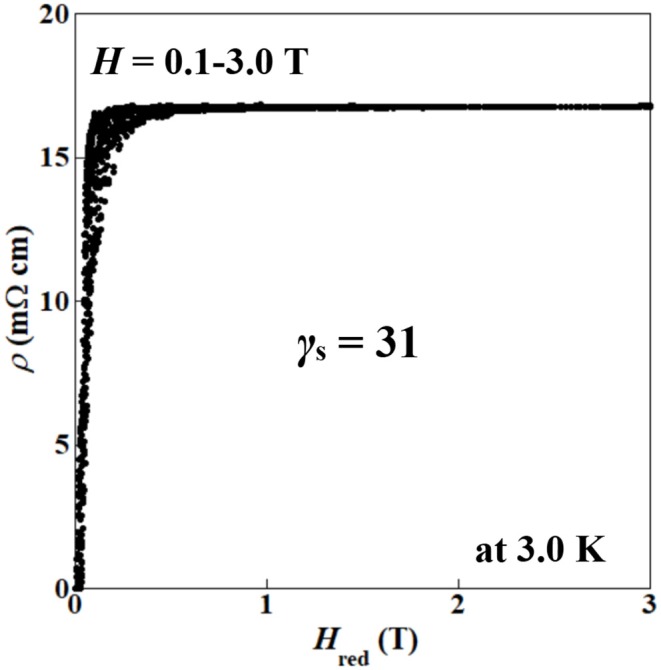
Reduced magnetic field *H*_red_ dependence of resistivity (ρ) scaling by Equation (1): $H_{\text{red}}=H{\left({\text{sin}}^{2}\theta +\gamma_{\text{s}}^{-2}{\text{cos}}^{2}\theta \right)}^{\text{1/2}}$ using Figure 10 data.

We also evaluated the superconducting anisotropy (γ_s_) of the (Ce,Pr)OBiS_2_ single crystal with *x* = 0.9 by an effective-mass model (Blatter et al., 1992). The angular (θ) dependence of resistivity (ρ) was measured under different magnetic fields (*H*) in the flux liquid state to estimate the superconducting anisotropy (γ_s_). The reduced field (*H*_red_) is calculated using the following equation for an effective mass model:

(1) $$\begin{array}{l} {\text{H}}_{\text{red}}=H{\left({\text{sin}}^{2}\theta +\gamma_{\text{s}}^{-2}{\text{cos}}^{2}\theta \right)}^{1/2} \end{array}$$

where θ is the angle between the *c*-plane and the magnetic field (Iye et al., 1992; Iwasaki et al., 1995). The γ_s_ was estimated from the best scaling of the ρ–*H*_red_ relationship. Figure 10 shows the θ dependence of ρ under different magnetic fields (*H* = 0.1–3.0 T) at 3.0 K in the flux liquid state for (Ce,Pr)OBiS_2_ single crystal with *x* = 0.9. The ρ–θ curve exhibited an almost two-fold symmetry. Figure 11 shows the ρ–*H*_red_ scaling obtained from the ρ–θ curves in Figure 10 using Equation (1). The scaling was performed by taking γ_s_ = 31. In contrast, the superconducting anisotropy (γ_s_) of (Ce,Pr)OBiS_2_ with other Pr concentrations (*x* = 0.3, 0.5, 0.7) single crystals using Equation (1) were estimated to be 7.5–13. These results suggest that the superconducting anisotropy of (Ce,Pr)OBiS_2_ single crystal with *x* = 0.9 is highest.

## Discussion

(Ce,Pr)OBiS_2_ single crystals with various Ce/Pr ratios were synthesized and characterized. All of the crystals exhibited tetragonal phase, and the superconducting transition temperature increased to 4 K with increasing ratio of Pr. With increasing Pr content, the lattice parameters of the *a*-axis decreased and those of the *c*-axis increased. TEM analysis revealed mixed occupancy of Ce and Pr without stacking faults.

The crystal structure and superconducting features found in the single crystals with high Pr concentrations were different from those of the corresponding powder samples reported previously (Miura et al., 2018). Single crystals obtained with flux exhibited tetragonal structure, whereas powders obtained without flux had a mixture of tetragonal and monoclinic structures. Although the single crystals with tetragonal structure showed the highest transition temperature at Ce_0.1_Pr_0.9_OBiS_2_ (*x* = 0.9), the corresponding powder sample with mainly monoclinic structure did not show zero resistivity (Miura et al., 2018). Therefore, although the analyzed compositions were similar, flux growth definitely affected the crystal structure and superconducting properties.

Superconducting transition temperature can be explained by carrier concentration and in-plane chemical pressure. The existence of Ce^3+^ and Ce^4+^ detected by X-ray absorption can provide carrier into the BiS_2_ layers. The increase in Ce^4+^ ratio found at high Pr content can compensate the decrease of Ce content. The increase in Ce^4+^ ratio showed a similar tendency as the increase in transition temperature (Figure 6). Considering the ionic radii (Ce^3+^ = 1.143 Å, Ce^4+^ = 0.97 Å, Pr^3+^ = 1.126 Å) (Shannon, 1976), increase in the ratio of Pr can decrease the lattice parameter of the *a*-axis. The decrease in *a*-axis can enhance the in-plane chemical pressure in the BiS_2_ plane, which would increase the superconducting transition temperature (Mizuguchi et al., 2015).

Ce_0.1_Pr_0.9_OBiS_2_ (*x* = 0.9) showed the highest superconducting transition temperature of ~4 K and also showed high superconducting anisotropy. When compared with Ce_0.3_Pr_0.7_OBiS_2_ (*x* = 0.7), the lattice parameter of the *a*-axis was slightly longer and that of the *c*-axis was significantly longer. The elongated *c*-axis could be the reason for the high superconducting anisotropy. The ratio of Ce^4+^/Ce^3+^ was the highest in the series, but this cannot explain the increase in the lattice parameters considering the ionic radii. TEM analysis did not show the ordering of Ce and Pr. Structural analysis using single crystal X-ray diffraction via a highly monochromatic synchrotron source would facilitate better understanding of the relationship between structure and superconductivity.

## Conclusion

Ce_1−*x*_Pr_*x*_OBiS_2_ (0.1 ≤ *x* ≤ 0.9) platelet single crystals were grown using a CsCl flux. The crystal structures of Ce_1−*x*_Pr_*x*_OBiS_2_ single crystals with 0.1 ≤ *x* ≤ 0.9 maintained the tetragonal phase. Increase in Pr content up to *x* = 0.7 decreased the lattice parameter of the *a*-axis and increased the parameter of the *c*-axis. Ce_0.1_Pr_0.9_OBiS_2_ single crystal showed a slightly elongated *a*-axis lattice parameter but a significantly elongated *c*-axis parameter. Atomic-resolution TEM analysis of the Ce_0.1_Pr_0.9_OBiS_2_ single crystal showed no stacking faults and no ordering of Ce and Pr. The superconducting transition temperature of Ce_1−*x*_Pr_*x*_OBiS_2_ single crystals increased with increasing Pr content, showing a trend similar to that of the ratio of Ce^4+^/(Ce^3+^ + Ce^4+^). High superconducting transition temperature of ~4 K and high superconducting anisotropy appeared in the Ce_0.1_Pr_0.9_OBiS_2_ (*x* = 0.9) single crystal. While the Ce_0.1_Pr_0.9_OBiS_2_ (*x* = 0.9) single crystal with tetragonal structure showed the highest transition temperature, the corresponding powder samples of mainly monoclinic phase did not show zero resistivity (Miura et al., 2018). Therefore, we believe that flux growth is a promising approach for exploring new superconductors.

## Data Availability Statement

The raw data supporting the conclusions of this article will be made available by the authors, without undue reservation, to any qualified researcher.

## Author Contributions

MN, AM, KT, and IT designed the research. MN and YMa prepared the samples. MN and SW measured electronic conductivity. MN and YT measured magnetic properties. MN and AM measured X-ray absorption. YG, YMi, CM, and YK measured SXRD. YW performed preliminary TEM analysis. DU and TA performed atomic-resolution TEM analysis. MN and AM wrote the draft. All the authors checked the manuscript.

### Conflict of Interest

The authors declare that the research was conducted in the absence of any commercial or financial relationships that could be construed as a potential conflict of interest.
